# Coculture of meniscus cells and mesenchymal stem cells in simulated microgravity

**DOI:** 10.1038/s41526-017-0032-x

**Published:** 2017-11-10

**Authors:** William M. Weiss, Aillette Mulet-Sierra, Melanie Kunze, Nadr M. Jomha, Adetola B. Adesida

**Affiliations:** 1 Laboratory of Stem Cell Biology and Orthopaedic Tissue Engineering, Department of Surgery, University of Alberta, Li Ka Shing Centre for Health Research Innovation, Edmonton, AB Canada; 2 Department of Orthopedic Surgery and Rehabilitation, Sports Medicine, Reconstruction and Trauma, Texas Tech University Health Sciences Center, Lubbock, TX USA

## Abstract

Simulated microgravity has been shown to enhance cartilaginous matrix formation by chondrocytes and chondrogenesis of mesenchymal stem cells (MSCs). Similarly, coculture of primary chondrocytes with MSCs has been shown as a strategy to simultaneously retain the differentiated phenotype of chondrocytes and enhance cartilaginous matrix formation. In this study, we investigated the effect of simulated microgravity on cocultures of primary human meniscus cells and adipose-derived MSCs. We used biochemical, qPCR, and immunofluorescence assays to conduct our investigation. Simulated microgravity significantly enhanced cartilaginous matrix formation in cocultures of primary meniscus cells and adipose-derived MSCs. The enhancement was accompanied by increased hypertrophic differentiation markers, *COL10A1* and *MMP-13*, and suppression of hypertrophic differentiation inhibitor, gremlin 1 (*GREM1*).

## Cartilage: Simulated microgravity promotes cartilage matrix deposition

Co-culture of meniscal cartilage-forming cells with fat-derived stem cells can lead to enhanced cartilage matrix production when cultured under simulated microgravity. Adetola Adesida from the University of Alberta in Edmonton, Canada, and colleagues cultured two types of cells found together in the knee—cartilage-forming chondrocyte cells (taken from the meniscus) and mesenchymal stem cells (isolated from the infrapatellar fat pad)—in a rotary cell culture system designed to model weightlessness on Earth. Simulated microgravity enhanced the synergistic interaction between the two types of cells in culture, resulting in more matrix production, but it also prompted the cartilage-forming cells to differentiate towards bone-forming cells, as evidenced by gene expression analysis. These findings suggest that microgravity and simulated microgravity-based culture technologies could help bioengineers grow knee replacements for people with meniscus tears, but increased bone-directed differentiation could pose a possible problem for astronauts on prolonged missions.

## Introduction

The menisci of the knee are a pair of fibrocartilaginous tissues.^1^ They primarily serve as mechanical load distributors within the knee joint.^2^ Traumatic tears in the avascular region of the tissue are common and do not heal.^1^ Treatment options for these tears are currently limited to partial meniscectomy.^3,4^ However, partial meniscectomy is a major risk factor for the early development of knee osteoarthritis.^3,4^ Tissue engineering using cells presents a potential option to create functional tissues to replace damaged meniscus.^5–15^ Primary meniscus cells (MC) are the ideal cell sources as they closely resemble the in vivo phenotype of MC and can form the biomechanically functional extracellular matrix (ECM) of the meniscus.^1,16,17^ Obtaining sufficient numbers of primary MC for meniscus tissue engineering is often impossible and impractical.^18,19^ To circumvent this limitation, primary MC are expanded in in vitro culture. However, expanded MC suffer from dedifferentiation and loss of the functional matrix-forming phenotype of native MC.^18,19^ Supplementation of primary MC through direct coculture with bone marrow mesenchymal stem cells (MSCs) has been demonstrated as a strategy to retain the differentiated phenotype of primary MC with the additional benefits of synergistic production of the functional matrix components of the meniscus^20–22^ and downregulation of hypertrophic differentiation of MSCs.^21,23^ However, the use of bone marrow MSCs potentially presents another anatomical site other than the knee for MSCs harvest. Supplementation of primary MC with knee infrapatellar fat pad derived MSCs may be a better anatomical option.

The differentiation of multiple types of MSCs is influenced by a wide range of biochemical,^24^ microenvironmental,^25^ and mechanical factors.^26–28^ TGF-β1 and TGF-β3, members of the TGF-β superfamily, that promote fibro-chondrogenic differentiation of MSCs^29^ and production of meniscus-like ECM. Diverse types of mechanical stimuli, including compressive load,^26^ simulated microgravity (SMG),^30^ low-intensity pulsed ultrasound,^27,28,31^ and hydrostatic pressure has been reported to induce or regulate the differentiation of MSCs through TGF-β signaling pathway.^32^ Hypoxia stimulated chondrogenic differentiation of bone marrow-derived MSCs by induction of TGF-β1 gene expression and protein production.^33^

The rotary cell culture system (RCCS) bioreactor, developed by the National Aeronautics and Space Administration (NASA), as a tool to simulate microgravity has been shown to provide a relatively well-defined fluid dynamic environment with efficient mass transfer for nutrients and gases and low shear stress for tissue growth.^34^ RCCS has been reported to produce hydrodynamic forces supporting the development of tissue structures resembling cartilage^35,36^ and meniscus.^37^ Simulated microgravity facilitated the retention of the differentiated phenotype of chondrocytes within 3D porous scaffolds.^36^ Moreover, RCCS synergistically enhanced TGF-β1-mediated chondrogenesis of human adipose-derived MSCs.^30^ However, to the best of our knowledge, no studies has investigated the effect of SMG on TGF-β-mediated chondrogenesis in cocultures of primary human MC and infrapatellar fat pad-derived MSCs.

In the present study, primary human MC were cocultured with infrapatellar fat pad-derived MSCs on a 3D porous collagen scaffold in the presence of TGF-β3, and cultured under SMG using the RCCS bioreactor. The goals of the present study were (a) to investigate the effect of SMG on human MC, (b) to determine the effect of SMG on the interaction of MC and MSCs in coculture and (c) to assess the potential benefit of using SMG to tissue engineer meniscus constructs in 3D porous type I collagen scaffolds. We hypothesized that SMG will augment the synergistic interaction between primary human MC and MSCs, and result in increased matrix production with suppression of hypertrophic differentiation.

## Results

### Colony forming characteristics and immuno-phenotype of adipose stem cells (ASC)

**Fig. 1:**
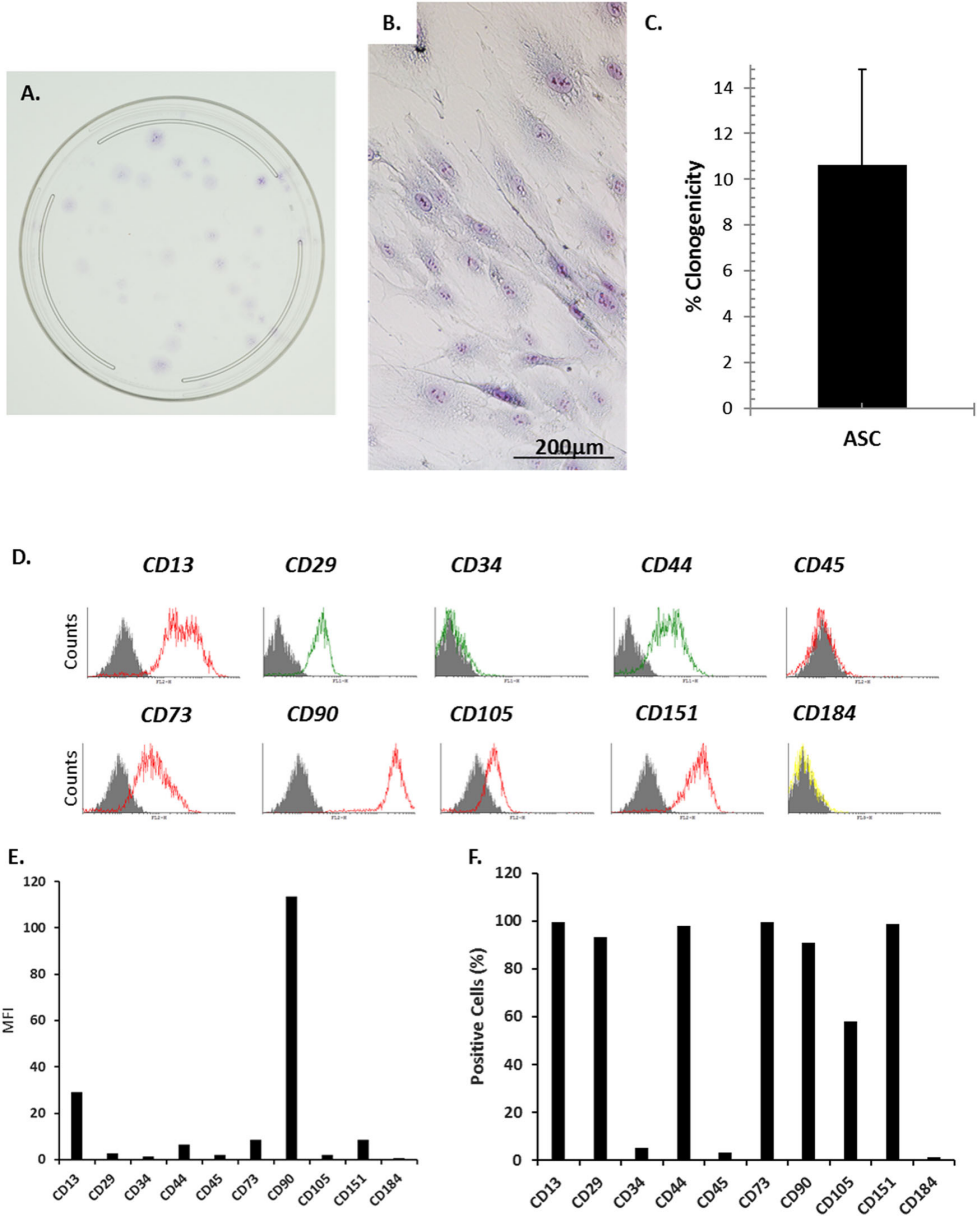
Colony-forming unit fibroblastic characteristic and surface marker phenotype of fat pad-derived cells following tissue culture plastic adherent culture expansion. **a** A digital photograph of a CFU-F assay Petri dish after staining with crystal violet. **b** Representative photomicrographs of adherent cells from a 40-year-old male at passage (P0). **c** Proportion (%) of total fat pad-derived MNCs seeded forming cell colonies (% clonogenicity). **d** Surface marker phenotype of fat pad-derived adherent cells following culture expansion until passage 2 (P2). For each CD marker tested, solid histograms show negative isotype control; open histograms show CD marker expression. **e** Represents the mean fluorescence intensity (MFI) of two donors. **f** Proportion of positive (%) cells expressing tested CD markers

A proportion of the seeded adipose tissue-derived mononucleated cells (MNCs) formed plastic adherent cell populations with distinct colonies. The developed colonies stained with crystal violet (Fig. 1a). Microscopic visuals of the colonies revealed cells with fibroblastic morphologies characteristics of plastic adherent MSCs (Fig. 1b). We determined the clonogenicity of the MNCs to be 10.65 ± 4.15% (Fig. 1c). The adherent cell populations were positive for the panel of cell surface markers characteristic of MSCs;^38,39^ CD13, CD29, CD44, CD73, CD90, CD105, and CD151 (Fig. 1d). Their relative mean fluorescence intensities (MFI) ± SD are as in the parentheses: CD13 (29.26 ± 10.79), CD29 (2.83 ± 0.35), CD44 (6.40 ± 2.40), CD73 (8.63 ± 3.79), CD90 (113.62 ± 14.90), CD105 (1.87 ± 0.05), and CD151 (8.57 ± 0.16)—Fig. 1e. The proportion (%) of cells that were positive for these markers varied considerably; CD13 (99.67 ± 0.15), CD29 (93.18 ± 3.26), CD44 (98.06 ± 0.08), CD73 (99.67 ± 0.09), CD90 (90.97 ± 8.86), CD105 (57.94 ± 11.38), and CD151 (98.86 ± 0.16), an indication of heterogeneity (Fig. 1f). In contrast to the MFI associated with mesenchymal markers, the cells displayed lower MFIs for markers of hematopoietic lineage; CD34 (1.29 ± 0.02) and CD45 (1.92 ± 0.50). The proportion (%) of cells that were positive for CD34 and CD45 were 4.93 ± 0.39 and 3.16 ± 1.10, respectively. Interestingly, the cells had a low MFI (0.73 ± 0.52) for the C-X-C chemokine receptor type 4 (CXCR-4) also known as CD184, which has been reported to be expressed on synovial fluid-derived MSCs.^40^ The proportion of CD184^+^ cells was 1.29 ± 0.05%.

### Fluorescent-labeled cells reveal coculture of primary MC and ASC

**Fig. 2:**
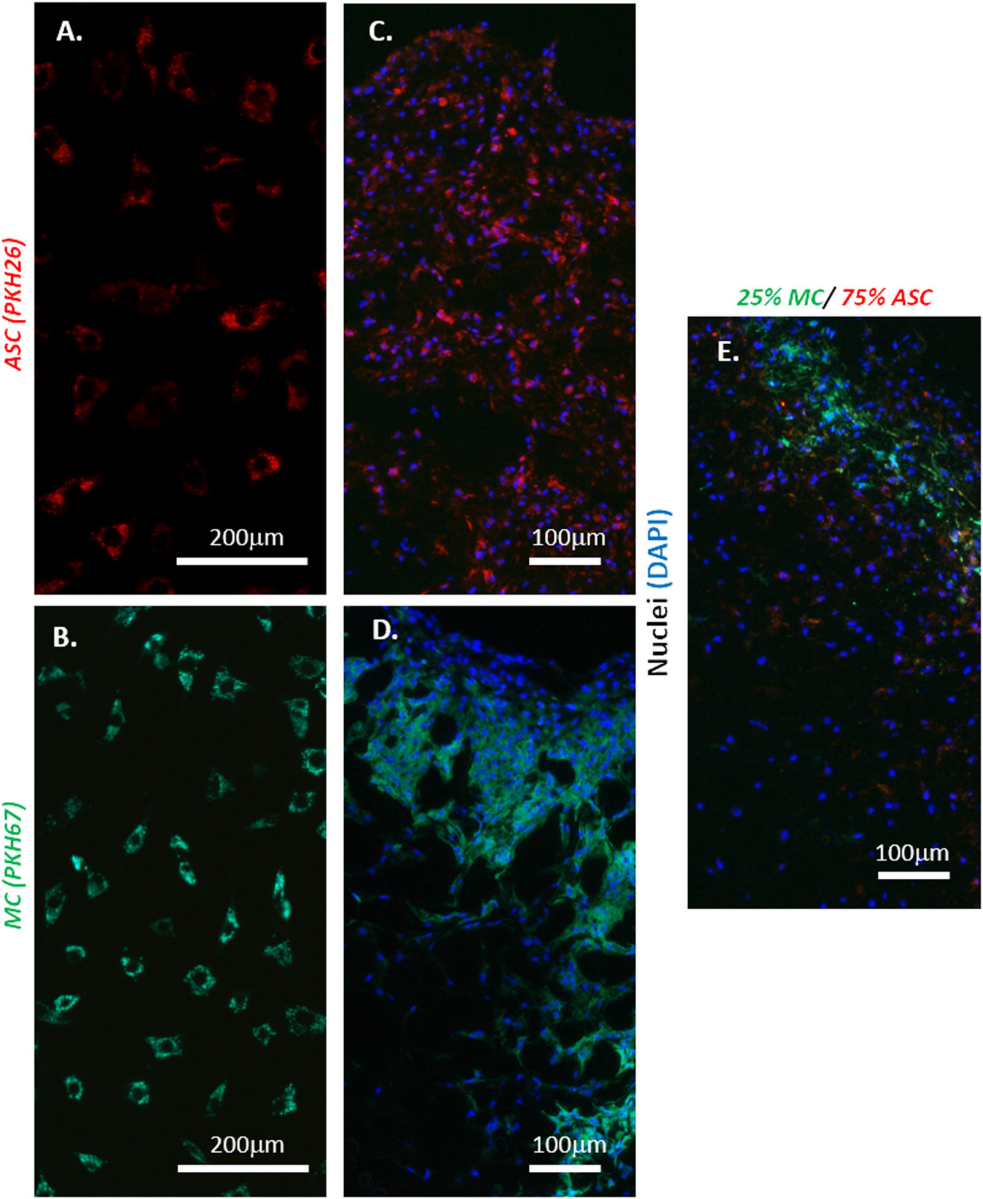
Fluorescence photomicrographs of PKH26 and PKH67 labeled cells in monolayer and in three-dimensional (3D) porous scaffolds. **a** Representative fluorescence photomicrograph of passage 2 (P2) fat pad-derived plastic adherent cells labeled with PKH26 (red) cell membrane dye (male, 19 years old). Cells were labeled in suspension and imaged after 30 min of plating in monolayer. **b** Representative fluorescence photomicrograph of primary human meniscus cells labeled with PKH67 (green) cell membrane dye (male, 56 years old). Cells were labeled in suspension and imaged after 30 min of plating in monolayer. **c** Representative fluorescence photomicrograph of passage 2 (P2) fat pad-derived plastic adherent cells (*ASC*) labeled with PKH26 (red) cell membrane dye (male, 19 years old) after 3 weeks of chondrogenic culture in porous type I collagen scaffold. Cells were visualized after sectioning at 7 μm and additional staining with DAPI for cell nuclei identification. **d** Representative fluorescence photomicrograph of primary human meniscus cells (*MC*) labeled with PKH67 (green) cell membrane dye (male, 56 years old) after 3 weeks of chondrogenic culture in porous type I collagen scaffold under static condition. Cells were visualized after sectioning at 7 μm and additional staining with DAPI for cell nuclei identification. **e** Representative fluorescence photomicrograph of cocultured primary human meniscus cells (*MC*) labeled with PKH67 (green) and passage 2 (P2) fat pad-derived plastic adherent cells (*ASC*) labeled with PKH26 (red) after 3 weeks of chondrogenic culture in porous type I collagen scaffold under static conditions. The cells were premixed at a cell ratio of 25% MC and 75% ASC prior to seeding a total of 250,000 cells per type I collagen scaffold. Cells were visualized after sectioning at 7 μm and additional staining with DAPI for cell nuclei identification. Scale bars: A and B (200 μm) and C–E (100 μm)

Pure ASC and pure MC were labeled successfully with cell membrane fluorescent dyes PKH26 (red; Fig. 2a) and PKH67 (green; Fig. 2b), respectively. Pure ASC (Fig. 2c), pure MC (Fig. 2d), and cocultured cells at 25% MC and 75% ASC (Fig. 2e) retained their respective fluorescent dyes after 4 weeks of chondrogenic culture in type I collagen matrix scaffolds. This aspect of the study was performed only under static conditions.

### Chondro-induction is enhanced in SMG cocultures of primary MC and ASC

**Fig. 3:**
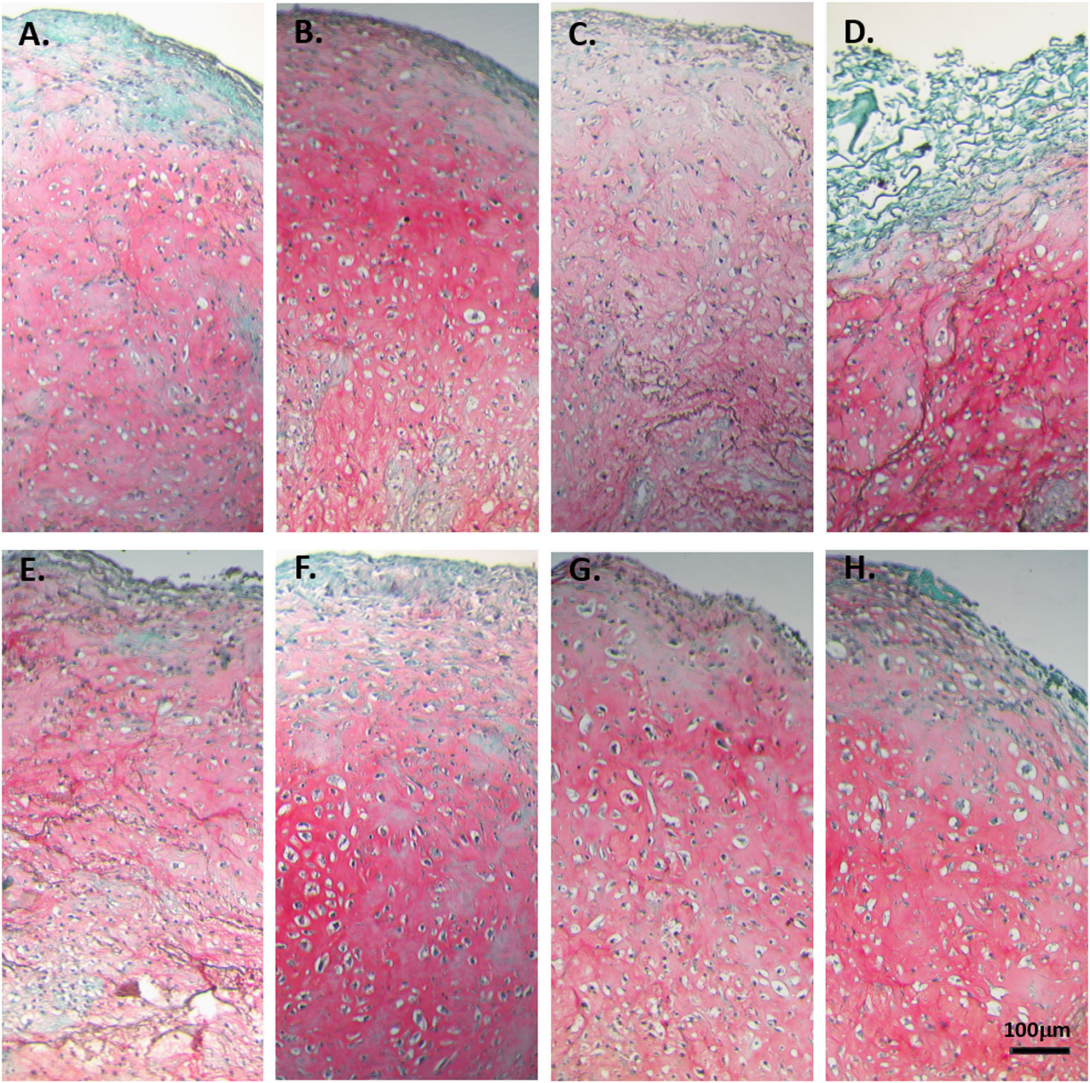
Histological Safranin O staining after chondrogenic stimulation and differentiation of primary human meniscus cells and fat pad-derived adipose stem cells in porous type I collagen scaffolds. Representative safranin O staining of cell-scaffold constructs after 4 weeks of culture in a defined serum-free chondrogenic medium containing transforming growth factor-β3 (TGFβ3) and dexamethasone (DEX) under *static* **a–d** and *simulated microgravity* **e–h** conditions: **a, e** pure primary human meniscus cells (MCs; male, 56 years); **b, f** Passage 2 (P2) fat pad-derived adipose stem cells (ASC; male, 19 years)**; c, g** Coculture of 75% (P2) fat pad-derived adipose stem cells (ASC; male, 19 years) with 25% primary human meniscus cells (MCs; male, 56 years); **d, h** Coculture of 50% (P2) fat pad-derived adipose stem cells (ASC; male, 19 years) with 50% primary human meniscus cells (MCs; male, 56 years). Scale bar 100 μm

**Fig. 4:**
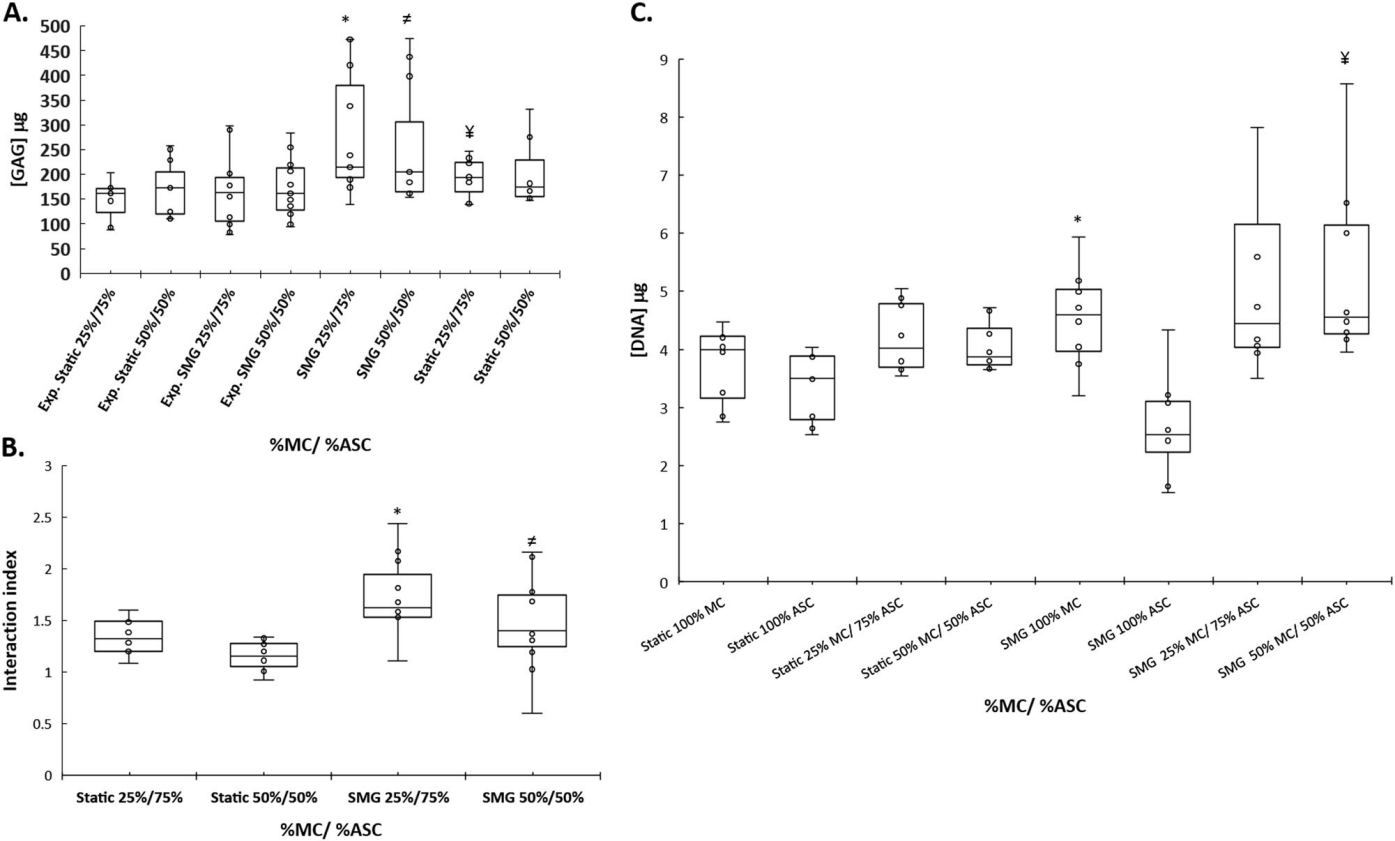
The interaction between primary human meniscus cells and fat pad-derived adipose stem cells on type I collagen scaffold after chondrogenic stimulation under static and simulate microgravity (SMG) conditions. Values are mean ± SD of four independent experiments. Expected GAG values are indicated with an “Exp.” prefix. The following symbols: *, ^≠^ and ^¥^ indicates statistically significant differences between expected and measured GAG values of cocultured group MC/ASC. **a** *Exp. SMG 25%/75% vs. SMG 25%/75%, ^≠^Exp. SMG 50%/50% vs. SMG 50%/50%, ^¥^Exp. Static 25%/75% vs. Static 25%/75%. **b** Interaction index values were determined from the ratio of measured and expected GAG as per the definition in section “Materials and methods”. Symbols *and ^≠^ indicate Wilcoxon signed rank test determined significant differences between the interaction indices of static and simulated microgravity (SMG) cocultured groups; *SMG 25%/75% vs. Static 25%/75% and ^≠^SMG 50%/50% vs. Static 50%/50%. **c** DNA contents of all groups after static and simulated microgravity culture conditions. Symbols * and ^¥^ indicate a paired one-tailed Student’s t-test (with unequal variance) statistically significant difference between the DNA contents of static and simulated microgravity (SMG) groups: *SMG 100% MC vs. static 100% MC, and ^¥^SMG 50%/50% vs. static 50%/50%

Cell-seeded scaffold constructs of pure MC, pure ASC, and cocultures of MC and ASC were cultured in serum-free chondrogenic media for up to 28 days in the presence of TGF-β3 under static or SMG conditions. Qualitative histological analysis of the constructs with safranin O staining demonstrated intense positive glycosaminoglycan (GAG) staining after the 28 days of culture under static and SMG conditions (Fig. 3). All groups were intensely positive for safranin O and nuclei content, an indication of significant synthesis and deposition of sulfated GAG matrix in the constructs (Fig. 3a–h). However, the deposition of GAG matrix appears to be more homogenously distributed in the constructs cultured under SMG (Fig. 3e–h). Except for pure MC constructs, the characteristic phenotype of hypertrophic chondrocytes of enlarged size/lacunae was evident in groups containing ASC. Quantitative analysis for the GAG contents of the constructs via DMB assay demonstrated that GAG contents were higher in constructs containing cocultured cells regardless of whether the constructs were cultured under static or SMG conditions (Fig. 4a). The constructs formed under static conditions from the coculture of 25% MC and 75% ASC had a measured GAG content of 195.25 ± 38.73 μg (mean ± SD) compared to an expected GAG content of 148.50 ± 38.36 μg. Wilcoxon signed rank pairwise comparison test between the measured and expected GAG quantities confirmed a significantly higher measured GAG content than the expected values (*p* = 0.003; Fig. 4a). Similarly, there were significant differences between the measured and expected GAG content of cocultured constructs containing 25% MC and 75% ASC after culture under SMG. The SMG cultured constructs had a measured GAG content of 279.39 ± 124.09 μg relative to an expected GAG content of 167.78 ± 75.39 μg. Pairwise comparison test using Wilcoxon signed rank test revealed a significant difference between the measured and expected GAG values (*p* = 0.003; Fig. 4a). After static culture, the constructs containing 50% MC and 50% ASC had a measured GAG content of 190.53 ± 47.84 μg. The expected GAG values for these constructs were 172.21 ± 54.80 μg. However, there was no significant difference between the measured and expected GAG values as per Wilcoxon signed rank test (*p* = 0.109; Fig. 4a). In contrast, after SMG culture, the constructs containing 50% MC and 50% ASC measured a significantly different GAG content of 251.63 ± 121.92 μg compared to an expected GAG content of 173.04 ± 62.29. The *p-*value for the pairwise comparison was 0.026 as per Wilcoxon signed rank test (Fig. 4a).

After calculating the interaction index as a ratio of the measured and expected GAG contents of the constructs containing cocultured cells, we confirmed that the interaction indices were greater than 1, signifying that chondro-induction had occurred (Fig. 4b). Shapiro Wilk test for normality of data distribution proved positive. The Levene’s test was significant and lacked homogeneity of variances. For cocultured cells at 25% MC and 75% ASC, the interaction index was 1.35 ± 0.16 after culture under static conditions and 1.72 ± 0.39 after culture under SMG (Fig. 4b). Paired one-tailed Student’s *t* test (with unequal variance) comparison of the interaction indices revealed a significantly higher chondro-induction under SMG conditions (*p* = 0.006; Fig. 4b). For cocultured cells at 50% MC and 50% ASC ratio, the interaction index was 1.16 ± 0.14 after static culture and 1.49 ± 0.44 after SMG culture. Similarly, the level of chondro-induction after SMG culture conditions was significantly higher (*p* = 0.022; Fig. 4b).

### Simulated microgravity increases DNA content in MC and cocultures

We next explored the possibility that cell proliferation may accompany the enhanced chondro-induction in cocultures of MC and ASCs after culture under SMG. Shapiro Wilk test for DNA content distribution proved to be normal. However, the Levene’s test for homogeneity of data variance proved negative. To that end, a paired one-tailed Student *t* test with unequal variance was used to assess differences between parallel static and SMG groups. First, we compared the DNA contents of constructs containing pure MC and pure ASC after static culture and SMG culture conditions. The DNA content of constructs containing pure MCs was 4.53 ± 0.81 µg after SMG culture compared to 3.73 ± 0.63 µg after static culture. These values were significantly different (*p* = 0.03; Fig. 4c). In contrast, the DNA content of constructs containing pure ASCs after static culture and SMG culture approached significant difference (*p* = 0.05). The DNA contents of pure ASC containing constructs after static culture and SMG culture were 3.35 ± 0.56 µg and 2.67 ± 0.84 µg, respectively (Fig. 4c). The constructs containing 25%MC and 75% ASC had a DNA content of 5.46 ± 2.11 µg after SMG culture and 4.20 ± 0.57 µg after static culture. These values were not significantly different (*p* = 0.08) (Fig. 4c). In contrast, constructs containing 50% MC and 50% ASC were significantly different (*p* = 0.03) in DNA contents after static culture and SMG culture. The constructs had a DNA content of 5.33 ± 1.49 µg after SMG culture and those after static culture had a DNA content of 4.06 ± 0.41 µg (Fig. 4c).

### Effect of SMG on gene expression of chondrogenically stimulated MC, ASC, and cocultures of MC and ASCs

We used quantitative polymearse chain reaction (qPCR) to investigate the gene expression of chondrogenically stimulated MC, ASC and cocultures of MC and ASCs after static and SMG culture conditions. Data followed normal distribution as assessed by Shapiro Wilk test. However, the Levene’ assessment of error variance proved highly significant. To this end, pairwise comparison between gene expression data of static and SMG cultured constructs were performed using paired Student’s *t* test with unequal variance.

**Fig. 5:**
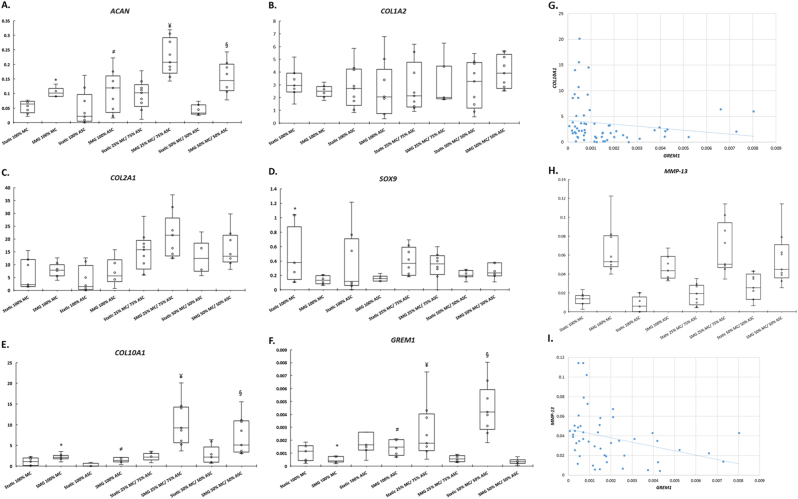
Gene expression of primary human meniscus cells, fat pad-derived adipose mesenchymal stem cells and cocultured cells under static and simulated microgravity culture conditions. Symbols *, ^≠^, ^¥^ and ^§^ indicate a paired Student’s *t* test (one-tailed; unequal variance) statistically significant differences between the interaction indices of static and simulated microgravity (SMG) cocultured groups; *SMG 100% MC vs. static 100% MC, ^≠^SMG 100% ASC vs. static 100% ASC, ^¥^SMG 25%/75% vs. static 25%/75% and ^§^SMG 50%/50% vs. static 50%/50%. **a** *ACAN*, **b** *COL1A2*, **c** *COL2A1*, **d** *SOX9*, **e** *COL10A1* and **f** *GREM1*. **g** Relative gene expression of *COL10A1* was plotted against the relative gene expression of *GREM1*. Pearson correlation analysis was used to assess the relationship between *COL10A1* and *GREM1*. **h** *MMP-13*. **i** Relative gene expression of *MMP-13* was plotted against the relative gene expression of *GREM1*. Pearson correlation analysis was used to assess the relationship between *MMP-13* and *GREM1*

**Fig. 6:**
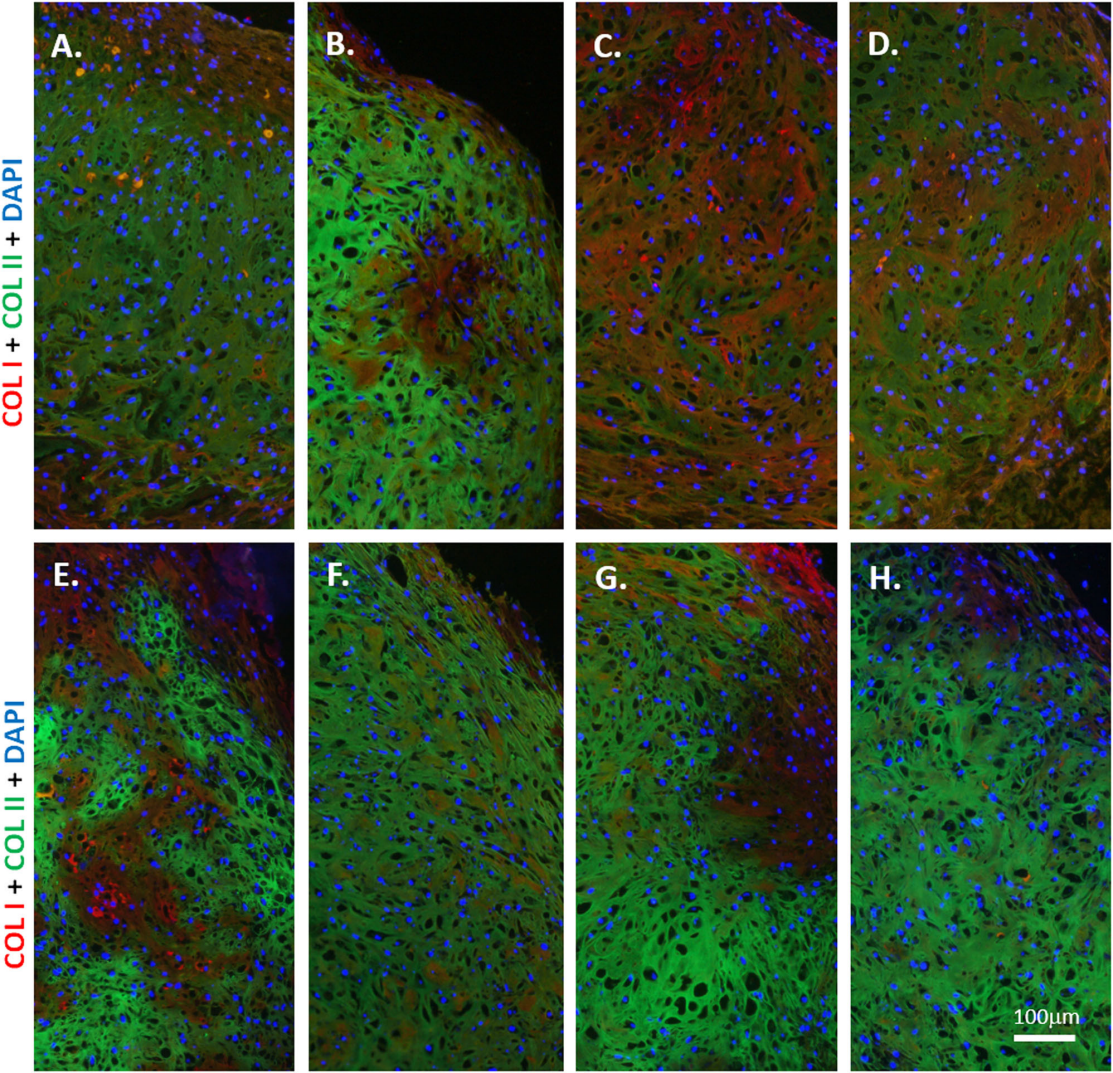
Immunofluorescence of types I and II collagen after chondrogenic stimulation and differentiation of primary human meniscus cells and fat pad-derived adipose stem cells in porous type I collagen scaffolds. Representative immunofluorescence of cell-scaffold constructs after 4 weeks of culture in a defined serum-free chondrogenic medium containing transforming growth factor-β3 (TGFβ3) and dexamethasone (DEX) under *static* **a–d** and *simulated microgravity* **e–h** conditions: **a, e** pure primary human meniscus cells (MCs; male, 56 years); **b, f** Passage 2 (P2) fat pad-derived adipose stem cells (ASC; male, 19 years)**; c, g** coculture of 75% (P2) fat pad-derived adipose stem cells (ASC; male, 19 years) with 25% primary human meniscus cells (MCs; male, 56 years); **d, h** Coculture of 50% (P2) fat pad-derived adipose stem cells (ASC; male, 19 years) with 50% primary human meniscus cells (MCs; male, 56 years). Scale bar 100 μm

SMG enhanced the mRNA expression of *ACAN* by 1.9-fold in pure MCs (*p* = 0.0002; Fig. 5a). SMG increased the mRNA expression of *ACAN* by 2.2-fold in pure ASC (*p* = 0.036; Fig. 5a). SMG enhanced the expression of *ACAN* by 2.4-fold in cocultures of 25% MC and 75% ASC (*p* = 0.0001; Fig. 5a). Similarly, SMG enhanced the mRNA expression of *ACAN* by 3.6-fold in 50% MC and 50% ASC cocultures (Fig. 5a). In contrast, the mRNA expression of *COL1A2* remained unperturbed between static and SMG cultured groups; no fold changes in *COL1A2* expression were observed. The insignificant differences in the treatment groups were supported by *p-*values > 0.05 (Fig. 5b). SMG increased the expression of *COL2A1* in pure MC, pure ASC, and in the cocultures of these cells. However, the fold increase observed were not statistically significant; SMG increased *COL2A1* mRNA expression by: 1.2-fold in pure MC (*p* = 0.28), 1.7-fold in pure ASC (*p* = 0.16), 1.4-fold in cocultures of 25% MC and 75% ASC (*p* = 0.06), and 1.2-fold in cocultures of 50% MC and 50% ASC (*p* = 0.21). Qualitative immunofluorescence detection confirmed the translation of these collagen genes in the constructs (Fig. 6). All groups were positive for type I and II collagen. No notable differences were evident in the deposition of these collagens between static culture and SMG culture conditions. All constructs treated with secondary antibodies only proved negative for the type I and II collagen (data not shown).

Interestingly, SMG significantly downregulated the mRNA expression of *SOX9* in pure MCs by 3.3-fold (*p* = 0.02; Fig. 5d). Similarly, SMG downregulated the expression of *SOX9* in pure ASCs and in cocultures of 25% MC and 75% ASC but not in coculture of 50% cells where a moderate increase of 1.2-fold was observed. These modulations in *SOX9* gene expression were not statistically significant with *p-*values >0.05.

SMG significantly enhanced the mRNA expression of the hypertrophic differentiation marker, *COL10A1* (Fig. 5e). The increase was two-fold in pure MC (*p* = 0.008), 7.8-fold in pure ASC (*p* = 0.03), 4.6-fold in cocultures of 25% MC and 75% ASC (*p* = 0.0009), and 2.7-fold in cocultures of 50% MC and 50% ASC (*p* = 0.01). We evaluated the gene expression of established inhibitor of hypertrophic differentiation, gremlin 1 (*GREM1*), to provide some mechanistic insight into the noted SMG-mediated upregulation of *COL10A1* (Fig. 5f). SMG downregulated the mRNA level of *GREM1* by two-fold (*p* = 0.02) in pure MC but not significantly in pure ASC (1.4-fold; *p* = 0.17). SMG significantly downregulated *GREM1* expression by 3.9-fold in cocultures of 25% MC and 75% ASC (*p* = 0.009), and by 12.4-fold in 50% MC and 50% ASC cocultures (*p* = 0.0001). Moreover, the mRNA expression of *COL10A1* and *GREM1* inversely correlated as assessed by Pearson correlation coefficient (−0.166). The correlation was not statistically significant (*p* = 0.100; one-tailed; Fig. 5g). In an analogous manner to collagen X, SMG increased the mRNA expression of *MMP-13*, another marker of hypertrophic differentiation of chondrocytes (Fig. 5h). It significantly increased the expression of *MMP-13*: in pure MC by five-fold (*p* = 0.0001), in pure ASC by five-fold (*p* = 1.78548 × 10^−6^), 3.7-fold in cocultures of 25% MC and 75% ASC (*p* = 0.0003), and 2.1-fold in coculture of 50% MC and 50% ASC (*p* = 0.008). Pearson correlation demonstrated a highly significant inverse relationship between the expression of *GREM1* and *MMP-13* (*r = *−0.305; *p* = 0.006; one-tailed; Fig. 5i).

**Fig. 7:**
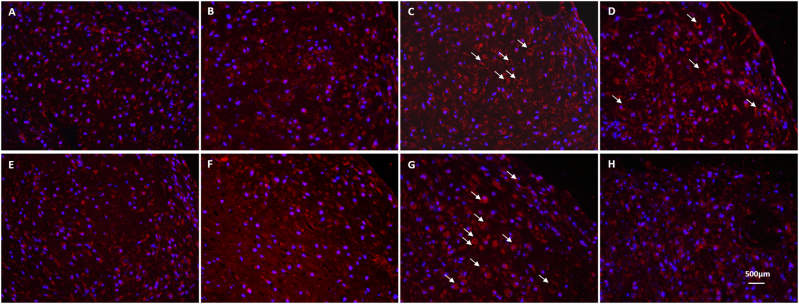
Immunofluorescence of type X collagen after chondrogenic stimulation and differentiation of primary human meniscus cells and fat pad-derived adipose stem cells in porous type I collagen scaffolds. Representative immunofluorescence of cell-scaffold constructs after 4 weeks of culture in a defined serum-free chondrogenic medium containing transforming growth factor-β3 (TGFβ3) and dexamethasone (DEX) under *static* **a–d** and *simulated microgravity* **e–h** conditions: **a, e** pure primary human meniscus cells (MCs; male, 56 years); **b, f** Passage 2 (P2) fat pad-derived adipose stem cells (ASC; male, 19 years)**; c, g** coculture of 75% (P2) fat pad-derived adipose stem cells (ASC; male, 19 years) with 25% primary human meniscus cells (MCs; male, 56 years); **d, h** coculture of 50% (P2) fat pad-derived adipose stem cells (ASC; male, 19 years) with 50% primary human meniscus cells (MCs; male, 56 years). Lacunae-like structures are indicated by white arrows. Scale bar 500 μm

We used immunofluorescence to confirm the translation of collagen X in the constructs (Fig. 7). Static culture of pure MC appears to have the least deposition of collagen X in the ECM (Fig. 7a). In contrast, the ECM of the pure MC after SMG culture appears to be more positive for type X collagen epitope (Fig. 7e). Similarly, SMG culture of pure ASC (Fig. 7f) appears to be more positive for type X collagen epitope than its static culture counterpart (Fig. 7b). Interestingly, in the cocultured constructs containing MC and ASC, the epitope of type X collagen was strikingly evident around structures (indicated by white arrows) characterized by a hypertrophic phenotype due to their enlarged size and lacunae-like appearance (Fig. 7c, d, g and h). These structures appear to be larger after SMG culture (Fig. 7g) relative to static culture (Fig. 7c), when MC and ASC cocultures are at a ratio of 25–75%, respectively. However, it is interesting to note that the presence of type X collagen epitope around the lacunae-like structures appear to be less evident in SMG cocultures of 50% MC and 50% ASC, but more so in the extracellular space (Fig. 7h) compared to their static cocultured counterparts (Fig. 7d). All constructs treated with secondary antibodies only proved negative for the type I and II collagen (data not shown).

## Discussion

The functional matrix of the knee meniscus is central to its biomechanical functionality within the joint. Generation of functional meniscus constructs, via tissue engineering and regenerative medicine strategies with a suitable cell source has the potential to revolutionize the treatment of avascular meniscus injuries, which do not heal. Avascular meniscus injuries account for most of meniscal lesions necessitating partial meniscectomies, which unfortunately predisposes the knee to early onset of osteoarthritis. Naturally, primary human MC are the optimal cell source for the generation of functional meniscal substitutes; however, limited availability from surgical specimen of partial meniscectomies is a drawback. Cell expansion strategies to increase numbers mitigate the expression of the functional matrix genes of the meniscus.^18,19^ To this end, direct coculture of primary human MC with adult-derived MSCs has been explored,^20,21^ to emulate the synergistic synthesis (i.e. chondro-induction) of the functional matrix of cartilage after coculture of primary articular or nasal chondrocytes with bone marrow or adipose MSCs.^41^ Simulated microgravity has been shown to synergistically enhance the synthesis of the functional matrix of articular cartilage in chondrocyte seeded polymeric scaffolds.^35,42^ Moreover, SMG synergistically enhanced the chondrogenic differentiation of adipose MSCs in the presence of TGFβ1.^30^ These reported findings prompted us to ask the following question: can SMG further augment chondro-induction in direct cocultures of primary human MC and adipose MSCs? We hypothesized that SMG will further enhance chondro-induction in primary human MC and adipose MSCs in the presence of TGFβ.

To investigate the hypothesis, human cells: adipose-derived MSCs and MC were successfully isolated from knee fat pad and menisci, respectively. Direct coculture of these cells in a 3D porous type I collagen matrix scaffolds, as a mimic of the predominant ECM of the native meniscus, was successfully demonstrated using differential fluorescent cell labeling. After 4 weeks of chondrogenic culture in the presence of TGFβ3, both pure cells and cocultured cells synthesized cartilaginous ECM under static and SMG.

Our study findings supported our hypothesis with evidence of GAG and *ACAN* increase. Moreover, SMG increased the DNA content of constructs containing pure MCs and cocultured MCs and ASCs. This was not the case in constructs containing pure ASCs. This finding suggested that the mechanism underlying SMG enhanced chondro-induction may involve increased proliferation of MC. Increased proliferation of chondrocytes in coculture with MSCs has also been reported previously as a mechanistic component of chondro-induction.^41^ An unexpected finding was the accompaniment of the microgravity enhanced chondro-induction with increased transcription of *COL10A1* and *MMP-13*, markers for hypertrophic chondrocytes and hypertrophic differentiation of MSCs.^43–48^ Previous work had demonstrated that direct coculture of primary^22^ or early passage^21^ human MC with MSCs suppressed hypertrophic differentiation of MSCs. The mechanism underlying this unexpected finding appears to be associated with the downregulation of *GREM1*, a known inhibitor/antagonist of hypertrophic differentiation.^49^ Parametric Pearson’s correlation coefficient was used to determine the strength of the association between *GREM1* and two hypertrophic markers: *COL10A1* and *MMP-13*. Our assessment revealed an inverse correlation (*r* = −0.166) between *COL10A1* and *GREM1*, albeit with a non-significant *p* value. There was a highly significant inverse correlation (*r* = −0.305, *p* = 0.006) between the expression of *MMP-13* and *GREM1*. The potential of increased hypertrophic differentiation through *GREM1* downregulation suggests a potential risk for enhanced bone formation in vivo through endochondral ossification pathway; conditional deletion of *GREM1* caused a transient increase in bone formation and bone mass.^50^ With that in mind, it is unclear at this point if outer and inner MC will behave differently in their capacity to modulate the expression of *GREM1* under SMG. Our previous work demonstrated that outer primary human MC have a greater capacity to suppress *COL10A1* and *MMP-13* expression.^22^ However, the study was conducted under static conditions using the cell pellet model of in vitro chondrogenesis. One may speculate that the bulk of primary human MC in this study may have originated from the inner region of the meniscus, since the inner portion of the tissue accounts for two-thirds of the meniscus’ width.

Our finding of increased *COL10A1* expression in pure adipose-derived MSCs under SMG is consistent with the reports of Yu et al.^30^ The authors demonstrated that SMG enhanced the in vitro chondrogenesis of adipose-derived MSCs in the presence of TGFβ1 but with increased *COL10A1* mRNA expression. They also demonstrated that SB203580, a highly specific and potent inhibitor of p38 mitogen-activated protein kinases (MAPK) signaling pathway, significantly suppressed the mRNA expression of *COL10A1* during SMG culture conditions, suggesting that p38 MAPK was a significant player in the transduction of physical forces mediated by SMG. Thus, activation of p38 MAPK signaling may be an additional underlying mechanism contributing to the synergistic chondro-induction in our study under SMG. It is noteworthy that while Yu et al.^30^ reported an increase in *COL2A1* and *SOX9* under SMG, we did not observe a significant increase in the expression of these genes under SMG. But our findings on *ACAN* and *COL10A1* transcriptional increase under SMG were consistent with the findings of Yu et al.^30^

In summary, the present study demonstrated that SMG enhanced the chondro-induction in cocultures of primary human MC and adipose-derived mesenchymal stem but at the cost of increased hypertrophic differentiation that is concomitant with *GREM1* downregulation.

## Methods

### Ethics statement

Approval of the Health Research Ethics Board-Biomedical Panel of the University of Alberta, Edmonton, Canada was obtained for this study, and institutional safety and ethical guidelines were followed. Ethics committee waived the need for written informed consent of patients, as specimens used in the study were intended for discard in the normal course of the surgical procedure. Extensive precautions were taken to preserve the privacy of the participants donating specimens.

### Isolation of primary human MC

Menisci were harvested from the knee joint of five donors: three males (14, 56, and 56) and two females (age 57 and 75), rinsed three times with sterile phosphate buffered saline (PBS), and finely minced. Donor information is documented in Table 2. MC were released from meniscus pieces by incubation for 16 hours at 37 °C in type II collagenase (0.15% w/v of 300 units/mg solid; Worthington, Lakewood, NJ, USA) in a standard medium, high glucose Dulbecco’s modified Eagle’s medium (DMEM) 4.5 mg/ml d-glucose supplemented with 5% v/v fetal bovine serum (FBS), 100 units/ml penicillin and 100 units/ml streptomycin, with l-glutamine (2 mM) (Invitrogen, Mississauga, Ontario, Canada), then filtered through a 150-µm nylon mesh to remove cellular debris, as described previously. Isolated cells were plated in standard medium supplemented in 150 cm^2^ tissue culture plastic (TCP) at 37 °C and 21% O_2_ in a humidified incubator with 5% CO_2_. After 48 h of recovery in standard medium, the cells were detached with 0.15% w/v trypsin-EDTA in Hank’s buffered saline solution (HBSS; Invitrogen) and counted prior to use.

### Isolation of human adipose stem cells

Patellar fat pads were harvested from the knee joint of five donors undergoing routine orthopedic procedure; three males (age 17, 19 and 40) and two females (age 20 and 21). Donor information is documented in Table 2. The adipose tissue was rinsed three times with PBS, and finely minced before enzymatic digestion. Tissue was digested with type II collagenase (0.15% w/v of 300 units/mg solid; Worthington) in PBS at 37 °C using a water bath shaker at 150 rpm for 45 minutes. Nucleated cells were re-suspended in alpha modified Eagle’s medium (αMEM) supplemented with 10% v/v FBS, and 1% w/v antibiotic/antimycotic solution filtered through a 100 μm nylon mesh to remove cellular debris. The number of nucleated cells in the aspirates was determined by crystal violet nuclei staining with cell counting using a hemocytometer. Thereafter, 15 million mono-nucleated cells (MNCs) were seeded per 150 cm^2^ TCP. The culture medium was αMEM supplemented with 10% v/v FBS, penicillin–streptomycin, 4-(2-hydroxyethyl)-1-piperazineethanesulfonic acid (HEPES), sodium pyruvate (all from Invitrogen) and 5 ng/ml basic fibroblast growth factor (bFGF or FGF2; from Humanzyme, Medicorp Inc., Montreal, Quebec, Canada) to maintain multipotency.^51–54^ The cells were cultured until passage 2 (P2) at 37 °C under 95% humidity in an atmosphere of 21% O_2_ and 5% CO_2_.

### Colony-forming unit fibroblastic (CFU-F) assay

A CFU-F assay was performed to determine the proportion of plastic-adherent cells from adipose-derived MNCs. MNCs from each donor were plated in triplicates at 500 MNCs per 100 mm diameter sterile Petri dish (Becton Dickinson Canada Inc.) and cultured for expansion as described above at 37 °C under 95% humidity in an atmosphere of 21% O_2_ and 5% CO_2_. After the first week, the non-adherent cell population was removed by aspiration and culture media was replenished twice for another week. After the 2 weeks of culture, the culture media was aspirated and the Petri dish was washed with sterile PBS, then fixed with 10% w/v buffered formalin for 5 minutes. The formalin was aspirated and the Petri dish was washed twice with PBS, and stained with 0.25% w/v crystal violet solution (Sigma-Aldrich). Stained colonies were revealed after washing with a copious amount of distilled water. Each stained cell collection was assessed and considered to be a colony as previously described.^55^ The number of colonies developed from the total number of MNC seeded was used to calculate clonogenicity (%), with each colony representing a single cell-derived clone.

### Flow cytometry analysis

All primary monoclonal antibodies used herein were directly conjugated antibodies to fluorescein isothiocynate (mAb-FITC) or to phycoerythrin (mAb-PE). Antibodies were either from BD Pharmingen or Invitrogen—see Table 1. The cells were analyzed on a FACScan flow cytometer (Becton Dickinson) after detachment from culture flask by 0.05% w/v trypsin-EDTA (Invitrogen). Staining buffer was prepared with PBS containing 1% w/v BSA (Sigma). The cells were re-suspended in 4 °C cold staining buffer at 5 × 10^6^/ml. Thereafter, the cells were dispensed into sample tubes (12 × 75 mm polystyrene round-bottom tubes, Becton Dickinson) in 20 µl aliquots and incubated for15 minutes with the antibodies at 4 °C. All incubations were implemented in 5 ml dilution tubes at room temperature in the dark and all washing steps were performed by a combination of centrifugation (400 g, 5 minutes) and aspiration of supernatant. Staining buffer (200 µl) was then added to the tubes and cells were incubated for 10 additional minutes. After removal of the supernatant by centrifugation, cells were washed with PBS and kept cold before analysis by flow cytometry. Non-specific staining was assessed using relevant isotype controls. Single color immuno-fluorescence analysis for the different surface markers was performed with mAb-FITC and mAb-PE. Data acquisition was performed with Cellquest software (Becton Dickinson). FITC emission was measured at FL1 and PE at FL3. For each sample a region for live cells was defined, according to the Forward Scatter (FSC) and Side Scatter (SSC) signals, which excluded aggregated cells from the analysis. Data analysis was performed with Cyflogic version 1.2.1, Perthu Terho & CyFlo Ltd., Finland. For each surface marker analyzed, percentage of positive cells and the level of marker expression were calculated. Percentage of positive cells was calculated as the percentage of cells having a measured fluorescence greater than that of 99.5% of the cells stained with each associated isotype control. Cells were considered positive for a surface marker when the percentage of positive cells for that surface marker was ≥6%. The level of expression of each marker was calculated as the ratio between geometric mean fluorescence intensity (MFI) of samples and that of the isotype control.

**Table 1:** Antibodies used to characterize infrapatellar fat pad mesenchymal stem cells

Specificity	Isotype*	Cat.#/Flurochrome	Source
CD13 (aminopeptidase-n)	mIgG1	sc-70529/PE	Santa Cruz Biotechnology
CD29	mIgG1	CD2901/FITC	Invitrogen
CD34	mIgG1	sc-19621/FITC	Santa Cruz Biotechnology
CD44 (Pgp-1, H-CAM, Ly 24)	mIgG2b	560977/FITC	BD Pharmingen
CD45 (2B11)	mIgG1	Sc-20056/PE	Santa Cruz Biotechnology
CD73	mIgG1	550257/PE	BD Pharmingen
CD90 (Thy-1)	mIgG1	55596/PE	BD Pharmingen
CD105 (endoglin)	mIgG1	sc-71043/PE	Santa Cruz Biotechnology
CD151 (PETA-3)	mIgG1	556057/PE	BD Pharmingen
CD184 (CXCR4)	mIgG2a	560937/PE-CyTM5	BD Pharmingen
Not specified (Isotype control)	mIgG1	sc-2855/FITC	Santa Cruz Biotechnology
Not specified (Isotype control)	mIgG1	sc-2866/PE	Santa Cruz Biotechnology

*m, mouse; r, rat

**Table 2:** Donor information of meniscal (MEN/ NMEN) and patellar fat pad (ASC) specimen

Donor	Age (years)	Gender (M/F)	Weight (kg)	Height (cm)	Smoker? (Y/N)	Medical history/surgery reason
MEN155	56	M	140	186	N	Osteoarthritis
MEN159	75	F	93	155	N	No anticancer drug use/osteoarthritis
MEN161	56	M	66	168	Y	No anticancer drug use/osteoarthritis
MEN172	57	F	96	151	N	Osteoarthritis
NMEN190	14	M	65	175	N	Partial meniscectomy
ASC64	20	F	–	–	–	Mitral valve prolapse/knee injury
ASC68	19	M	69.5	173	–	Healthy/meniscal & ACL repair
ASC67	17	M	79.5	185	N	Knee joint injury
ASC70	21	F	71	161	Y	Allergy to nickel, asthma
						Knee repair
ASC97	40	M	88.9	180	Y	Multiple ligament & knee injury

### Type I collagen scaffold constructs

Type I collagen matrix (Integra Lifesciences, PlainsBoro, NJ, USA; 10 cm × 12.5 cm; 3.5 mm total thickness collagen sponge with pore size of 115 ± 20 µm) was cut into 6 mm diameter disks using a sterile biopsy punch. To limit variability between batches of scaffolds, disks taken from the same batch of scaffolds were used for each experiment. The disks were placed in a 24-well plate for seeding with cells.

### Co-culture of MCs and ASCs

Cells were co-cultured in 25%:75% and 50%:50% ratios of MCs to ASCs, in addition to controls of each cell type alone. MCs and ASCs were sex matched, but obtained from different donors. After counting, cells were combined in the required ratios, and were carefully seeded on the Type I collagen scaffold via a micropipette with a total of 250,000 cells suspended in 20 µl of a defined serum-free chondrogenic medium consisting of high glucose DMEM containing 0.1 mM non-essential amino acids, 1 mM sodium pyruvate, 100 mM HEPES buffer, 1 mM sodium pyruvate, 100 U/ml penicillin, 100 mg/ml streptomycin, 0.29 mg/ml l-glutamine (Invitrogen) supplemented with 0.1 mM ascorbic acid 2-phosphate, 40 mg/ml l-proline, 10^−7^ M dexamethasone, 1 × ITS + 1 premix (Sigma-Aldrich, Oakville, Canada), 10 ng/ml TGF-β3 (Humanzyme-Medicorp Inc.). The seeded disks were transferred to a humidified incubator at 37 °C with 21% O_2_ and 5% CO_2_ for 15 minutes to allow initial cell attachment. Thereafter, 100 µl of chondrogenic medium was gently added to the base of each well containing cell-seeded disks followed by a 30 minutes incubation period in a humidified incubator at 37 °C with 21% O_2_ and 5% CO_2_. After the incubation period, 700 µl of chondrogenic medium was added slowly to the base of each well until the entire seeded scaffolds were covered. The seeded scaffolds were then incubated at 37 °C with 21% O_2_ and 5% CO_2_ for 7 days. Chondrogenic media exchange was performed twice per week.

### Co-culture of fluorescently labeled MCs and ASCs

To demonstrate co-culture of MCs and ASCs, isolated MCs and ASCs were labeled with fluorescent dyes in suspension according to manufacturer’s labeling protocol. Pure MCs were labeled with PKH67 (green) and pure ASCs were labeled with PKH26 (red). The labeled cells were visualized in monolayer after plating in a T25 TCP flask using an Eclipse Ti–S microscope (Nikon Canada Mississauga, Canada) fitted with NIS Elements (version 4.20; Nikon Canada). PKH67 and PKH26 fluorescent cell linker kits were purchased from Sigma-Aldrich (Oakville, Canada). The labeled cells were seeded onto type I collagen matrix scaffolds as described above. Culture media, conditions, and duration were as described above but only static culture was implemented to demonstrate co-culture of labeled cells. Cells were mixed at a ratio of 25% MC and 75% ASC prior to seeding on type I collagen matrix scaffold. Cell-scaffold constructs were cultured in chondrogenic medium as before for 28 days. Thereafter, the cultured constructs were embedded in a VWR clear frozen section compound (VWR, Mississauga, ON, Canada). Engineered tissue sections were cut on a Leica Biosystems cryostat at 7 µm. Images were captured as before and assembled in Image J version 1.51k software (NIH, Bethesda, Maryland, USA).

### Culture in SMG

After 7 days of culture, one set of cell co-culture on type I collagen scaffolds (MC alone, ASC alone, 1:3 MC to ASC, and 1:1 MC to ASC) were transferred to 10 mL vessels and inserted into a rotary bioreactor RCCS (Synthecon, Inc., Houston, TX). An identical set of co-cultures was used as a control, and continued to be incubated under static conditions. The rotary bioreactor was housed within the same incubator as the static controls, and both continued to be cultured in a humidified incubator at 37 °C with 21% O_2_ and 5% CO_2_. The rotation speed of the RCCS increased from 20 to 30 rpm over the course of 21 days of culture. This increase in rotation speed was required to keep the increasing mass of the scaffolds suspended under microgravity conditions. After 21 days of culture in the rotary bioreactor (28 days total from seeding of the scaffolds), the culture scaffolds were harvested and analyzed. Scaffolds were processed biochemically for GAG and DNA content, histologically and immunofluorescence for cartilage-specific matrix proteins, and at the molecular level by real time qPCR for gene expression analysis.

### Biochemical analysis

After culture, scaffolds were rinsed in PBS (Invitrogen), and frozen at −80 °C. For analysis, samples were digested in proteinase K (1 mg/ml in 50 mM Tris with 1 mM EDTA, 1 mM iodoacetamide and 10 mg/ml pepstatin A—all from Sigma-Aldrich) for 16 h at 56 °C. The sulphated GAG content was measured by 1,9 dimethylmethylene blue (DMB) binding (Sigma-Aldrich) using chondroitin sulfate (Sigma-Aldrich) as standard. The DNA content was determined using the CyQuant cell proliferation assay kit (Invitrogen) with supplied bacteriophage λ DNA as standard. Chondro-induction is defined as previously described.^41^ Based on experimentally measured GAG contents of pure MC seeded scaffolds (GAG_100% MCs_) and pure ASC seeded scaffolds (GAG_100% ASC_), the expected total GAG (GAG_expected_) in the coculture cell-seeded scaffolds was calculated as a linear function of the proportion (%) of MC using the following equation:

$${\mathrm{GAG}}_{{\rm{expected}}} = {\rm{GAG}}_{100{\rm{\% }}\,{\rm{MC}}} + \left( {{\rm{GAG}}_{100{\rm{\% }}\,{\rm{MC}}}-{\rm{GAG}}_{100{\rm{\% }}\,{\rm{ASC}}}} \right) \times {\rm{\% }}\,{\rm{MC}}$$

The interaction index was then calculated as the ratio of the GAG measured in the cocultured cell-seeded scaffolds (GAG_measured_) to the GAG_expected_. When the interaction index is higher than 1, then chondro-induction is considered to have occurred.^20,41^

### Histology and immuno-histochemistry

Tissues generated from the scaffold-cell cultures were fixed in 4% v/v phosphate buffered formalin, processed into paraffin wax, sectioned at 5 µm and stained with safranin O and counterstained with fast green, to reveal sulfated proteoglycan (GAG) matrix depositions. Other sections were probed with antibodies raised against collagen types I and II. The distribution of types I and II collagen were evaluated using immunofluorescence imaging. Sections were deparaffinized and rehydrated. Antigen retrieval was conducted with Protease XXV (Thermo Scientific) for 30 minutes at room temperature and hyaluronidase (H6254, Sigma) for 30 min at 37 °C. Sections were then blocked with 5% w/v bovine serum albumin in PBS for 30 minutes at room temperature. Collagen I, II and X labeling was completed using rabbit anti-collagen I, 1:200 dilution (CL50111AP-1, Cedarlane Labs, Burlington, ON, Canada), mouse anti-collagen II, 1:200 dilution (II-II6B3, Developmental Studies Hybridoma Bank, University of Iowa, USA) and rabbit anti-collagen X (ab58632, Abcam, UK) using a 1:100 dilution, respectively, and incubated overnight at 4 °C. Secondary labeling using goat anti-mouse IgG, Alexa Fluor 488, 1:200 (ab150117, Abcam) and goat anti-rabbit IgG, Alexa Fluor 594, 1:200 (ab150080, Abcam) incubated at room temperature for 1 hour. Slides were mounted using Everbrite Hardset Mounting Medium with DAPI (Biotium) and imaged using a Nikon Ti–S Microscope fitted with DS-U3/Fi2 Color CCD camera using FITC and Texas Red filters (Nikon Canada Mississauga, Canada) with NIS Elements software (version 4.20; Nikon Canada) and assembled in Image J version 1.51k software (NIH, Bethesda, Maryland, USA).

### Gene expression analysis

Total RNA was extracted from MCs, ASCs, and scaffold cultures using Trizol (Invitrogen) after grinding with Molecular Grinding Resin (Geno Technology Inc., St Louis, USA) in combination with the use of RNeasy mini-kit (Qiagen, Mississauga, ON, Canada) and after removal of contaminating genomic DNA from the preparations with DNase treatment. Total RNA (100 ng) in a 40 µl reaction was reverse transcribed to cDNA using GoScript reverse transcriptase (Fisher Scientific, Whitby, Ontario, Canada) primed with random primer oligonucleotides. qPCR was performed either in a DNA Engine OpticonIContinuous Fluorescence Detection System (Bio-Rad) using hot start Taq and SYBR Green detection (Eurogentec North America Inc, San Diego, CA, USA) or in a Bio-Rad CFX connect real-time system (Bio-Rad Laboratories, Mississauga, ON, Canada). Primer sequences were either designed by Primer Express 3.0.1 (Applied Biosystems, ThermoFisher, ON, Canada) or taken from previously published work. All primers were obtained from Invitrogen, Mississauga, ON, Canada. Gene (mRNA) expression levels for each primer set were normalized to the expression level of human βactin, by the 2-^ΔΔct^ method. Primer sequence are as follows: *ACAN* ^19^ (Accession# M55172) forward: AGG GCG AGT GGA ATG ATG TT; *ACAN* ^19^ reverse: GGT GGC TGT GCC CTT TTT AC; *ACTB* ^19^ (Accession# NM_001101) forward AAG CCA CCC CAC TTC TCT CTA A; *ACTB* ^19^ reverse AAT GCT ATC ACC TCC CCT GTG T; *COL1A2* ^19^ (NM_000089) forward: TTG CCC AAA GTT GTC CTC TTC T; *COL1A2* ^19^ reverse: AGC TTC TGT GGA ACC ATG GAA; *COL2A1* ^19^ (Accession# NM_033150) forward CTG CAA AAT AAA ATC TCG GTG TTC T; *COL2A1* reverse: GGG CAT TTG ACT CAC ACC AGT; *COL10A1* (Accession# X60382) forward: GAA GTT ATA ATT TAC ACT GAG GGT TTC AAA; *COL10A1* reverse: GAG GCA CAG CTT AAA AGT TTT AAA CA; *GREM-1* (Accession# NM_001191322.1) forward: CAT GTG ACG GAG CGC AAA TA; *GREM-1* reverse: GCT TAA GCG GCT GGG TTT T; *MMP13* (Accession# NM_002427) forward: CATCCAAAAACGCCAGACAA; *MMP13* reverse: CGGAGACTGGTAATGGCATCA; *SOX9* ^19^ (Accession# Z46629) forward: CTT TGG TTT GTG TTC GTG TTT TG; *SOX9* ^19^ reverse: AGA GAA AGA AAA AGG GAA AGG TAA GTT T.

### Statistical analysis

A total of five independent experiments were performed with five donor specimens for each cell type. Unless stated otherwise, numerical data distribution represents data from five donors and is presented as a boxplot of the minimum, first quartile, median, third quartile, and maximum values. Statistical analyses were performed using SPSS (version 23; IBM Canada Ltd, ON, Canada). Outliers were defined as values 1.5 interquartile ranges below the first quartile or above the third quartile. Data were assessed for normality using the Shapiro–Wilk test. Levene’s test was used to assess homogeneity of error variances. Statistical test choice was dependent on outcome of normality and homogeneity test. Paired comparisons between static and SMG groups was analyzed using either Wilcoxon signed rank test or paired Student’s *t* test depending on outcome of normality test. Pearson or Spearman correlation coefficient was used to assess correlation between genes. Statistical significance was considered when *p* < 0.05.

### Data availability

All relevant data are available from the corresponding author.
